# Cyclic Synthetic Peroxides Inhibit Growth of Entomopathogenic Fungus *Ascosphaera apis* without Toxic Effect on Bumblebees

**DOI:** 10.3390/molecules25081954

**Published:** 2020-04-22

**Authors:** Ivan A. Yaremenko, Mikhail Y. Syromyatnikov, Peter S. Radulov, Yulia Yu. Belyakova, Dmitriy I. Fomenkov, Vasily N. Popov, Alexander O. Terent’ev

**Affiliations:** 1 N. D. Zelinsky Institute of Organic Chemistry, Russian Academy of Sciences, Moscow 119991, Russia; yaremenko@ioc.ac.ru (I.A.Y.); radulov.peter@mail.ru (P.S.R.); beljulka@inbox.ru (Y.Y.B.); cpl.fom@gmail.com (D.I.F.); 2 All-Russian Research Institute for Phytopathology, B. Vyazyomy, Moscow Region 143050, Russia; 3 Department of Genetics, Cytology and Bioengineering, Voronezh State University, Voronezh 394018, Russia; syromyatnikov@bio.vsu.ru (M.Y.S.); pvn@bio.vsu.ru (V.N.P.); 4 Voronezh State University of Engineering Technologies, Voronezh 394036, Russia

**Keywords:** peroxide, ozonide, tetraoxane, fungicidal, *Ascosphaera apis*, fungi

## Abstract

In recent years, the number of pollinators in the world has significantly decreased. A possible reason for this is the toxic effects of agrochemicals reducing the immunity of insects that leads to their increased susceptibility to pathogens. *Ascosphaera apis* is a dangerous entomopathogenic fungus, afflicting both honeybees and bumblebees. We investigated fungicide activity of cyclic synthetic peroxides against *A. apis* isolated from *Bombus terrestris* L. The peroxides exhibited high mycelium growth inhibition of *A. apis* up to 94–100% at concentration 30 mg/L. EC_50_ values were determined for the most active peroxides. Two peroxides showed higher antifungal activity against *A. apis* than the commercial fungicide Triadimefon. The studied peroxides did not reduce the ability of bumblebees to fly and did not lead to the death of bumblebees. A new field of application for peroxides was disclosed.

## 1. Introduction

Organic peroxides are an important and attractive class of compounds for the development of bioactive agents. It has been shown that peroxides possess antimalarial [1,2,3,4,5,6], antiparasitic [7,8,9,10,11], anticancer [12,13,14,15,16,17], antiviral [18,19,20], anti-tuberculosis [21,22,23] activities. In 2015, the Nobel Prize in Medicine was awarded to Youyou Tu for the discovery and development of Artemisinin, a natural peroxide antimalarial drug [24]. Recently, we have discovered a new class of antifungal agents for crop protection, cyclic peroxides: bridged 1,2,4,5-tetraoxanes and bridged ozonides [25]. These peroxides possess a high fungicidal activity against a broad spectrum of phytopathogenic fungi.

Pollination plays a crucial role in the maintenance of biodiversity. There is a lot of evidence of recent declines in the population of both wild and tamed pollinators [26,27,28,29]. The decrease in the number of bumblebees and honeybees has already become a matter of food security because insect pollination is obligatory for obtaining a harvest of entomophilic crops consumed by humans [30]. One possible reason for this is the toxic effects of agrochemicals [31,32]. The search for fungicides, which can effectively kill various fungal pathogens without harming beneficial insects, such as honeybees and bumblebees, is urgent. Organic peroxides can be successful candidates for this role.

Studies of the effects of agrochemicals on insects are mainly concerned with assessing their effects on mortality, fertility, and physiological parameters. Recently, significant attention has been focused on the study of the negative impact of neonicotinoid pesticides on bumblebees and bees. For example, it has been shown that Thiamethoxam has reduced motor activity of bees [33], as well as the duration and distance of a flight [34]. It has also been revealed that neonicotinoids could reduce the viability of bumblebee queens and their nesting activity [35]. Neonicotinoids have been demonstrated to act synergistically with other stress factors, such as poor nutrition [36].

Pesticides that are not insecticides (fungicides, herbicides, etc.) can also negatively affect pollinators. Much less attention is paid to the toxic effect of these compounds on the physiological parameters of honeybees, bumblebees, and other beneficial insects. It has been noted that fungicides have a synergistic effect when they act simultaneously with neonicotinoids [37,38,39,40]. A significant correlation has been found between fungicide exposure and bee family collapse [31]. In honeybees that are exposed to fungicides, ATP production is reduced [41]. Using high-performance sequencing, it has been shown that a possible mechanism for the negative effect of fungicides on bees is their effect on insect microbiomes [42]. Under the action of fungicides, a significant change occurs in the quantitative and species composition of bacteria in the intestines of bees [43]. We previously showed that fungicides were able to inhibit the respiration of the mitochondria of bumblebee flight muscles [44].

Recently, it has been suggested that the synergistic effects of pesticides and diseases that affect pollinators contribute to a drastic reduction in the number of insect pollinators [45,46,47,48]. Pesticides reduce the overall immunity in insects that leads to their increased susceptibility to pathogens. Fungal pathogens are distinguished among the pathogens of the most important pollinators, and *Ascosphaera apis* (Maassen ex Claussen)—the causative agent of ascospherosis—represents one of them. The disease caused by this pathogen is contagious and destructive for honey bees [49]. *A. apis* causes losses in both bee population and productivity [50]. The larvae of honeybees are mainly infected with this pathogen when the sexual spores of *A. apis* are ingested. After that, the spores germinate in the intestinal lumen [51]. In addition, this pathogen has been found in bumblebees [52]. It has been assumed that bumblebees are only a carrier of this pathogen. However, recently, it has been reported that *A. apis* could cause bumblebee disease [53,54].

**Figure 1:**
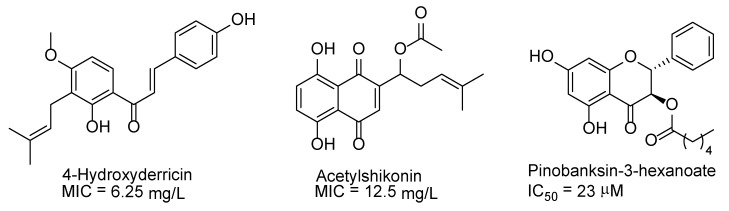
Structures of natural compounds with fungicidal activity against *A. apis*.

The development of fungicides against *A. apis*, effective and non-toxic to pollinators, humans, and the environment, is a complex task. Taking these requirements into account, the search for fungicides against *A. apis* has been based mainly on natural products. It has been found that 4-hydroxyderricin—one of the phytochemical components of plant *Angelica keiskei* and *Acetylshikonin* containing in the roots of *Lithospermum erythrorhizon*—is active against *A. apis* (MIC = 6.25 and 12.5 mg/L, respectively) (Figure 1) [54]. Pinobanksin-3-hexanoate isolated from propolis has demonstrated IC_50_ = 23 ± 2 μM [55]. However, the application of natural compounds is characterized by such disadvantages, such as low availability and high cost. Developing effective synthetic fungicides against *A. apis* can decide these problems.

In this paper, we investigated the effect of cyclic peroxides against the fungal pathogen of bumblebees and honeybees—*A. apis*. The toxicity of peroxides on bumblebees was also evaluated. Here, we disclosed a new field of application for peroxides.

## 2. Results

### 2.1. Identification and Isolation of Ascosphaera apis

We carried out a search of eukaryotic pathogenic microorganisms in larvae of bumblebees. To do this, the contents of the intestines of bumblebees were seeded onto the Saburo medium. After that, all grown colonies were isolated into separate Petri dishes and reared for 2 days. Further, DNA was extracted from the grown colonies (or mycelium) of microorganisms and sequenced with universal primers for fungi. Sequences were compared with those already available in NCBI GenBank and BoldSystem databases. As a result, the following eukaryotic microorganisms were detected in the intestines of bumblebee larvae: *Lachancea thermotolerans* (99.85% identity in NCBI GenBank), *Mucor racemosus* (99.84% identity in NCBI GenBank), *Naganishia adeliensis* (99.36% identity in NCBI GenBank), *Penicillium commune* (97.39% identity in NCBI GenBank), *Rhodotorula mucilaginosa* (100.00% identity in NCBI GenBank), *Ascosphaera apis* (99.36% identity in NCBI GenBank) (see in the Supplementary Materials).

The most dangerous identified microorganism for bumblebees and bees was *Ascosphaera apis*. This microorganism was isolated into individual Petri dishes. Further testing of synthetic peroxide was carried out with this microorganism.

### 2.2. The Effect of the Peroxides on the Inhibition of Growth of Ascosphaera apis

**Figure 2:**
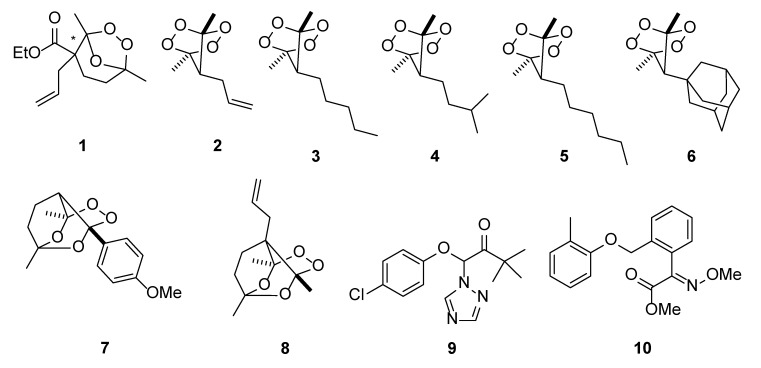
Structures of investigated peroxides **1**–**8** and the commercial fungicides Triadimefon **9** and Kresoxim-methyl **10**.

All peroxides **1**–**8**, shown in Figure 2, were evaluated for the inhibition of the radial growth of *Ascosphaera apis* mycelium in potato-saccharose agar. Triadimefon **9** and Kresoxim-methyl **10** were tested as the control compounds. They are widely used in agrochemistry as the broad-spectrum fungicides.

The results showed that ozonide **1** and tetraoxanes **2**, **3**, and **6** exhibited strong fungicidal activities, which exceeded the activities of fungicides Triadimefon **9** and Kresoxim-methyl **10** at a concentration of 30 mg/L (Table 1).

The mycelium growth inhibition (I) rates were calculated with the following equation: I = [(DC − DT)/DC] × 100%. Where DC is the control settlement diameter (mm), and DT is the treatment group fungi settlement diameter (mm). Ozonide **1** and tetraoxane **6** demonstrated a high inhibition of the mycelium growth (94%) of *Ascosphaera apis*. In the case of tetraoxanes **2** and **3**, the result was very impressive. The mycelium growth inhibition of *Ascosphaera apis* was 100%. Bridged 1,2,4,5-tetraoxanes **4** and **5** and monoperoxide **7** exhibited moderate activity (I = 49–66%). Unfortunately, monoperoxide **8** demonstrated low activity (I = 25%).

Further, for the most active peroxides (**1**–**3**, **6**) and reference compounds, we determined EC_50_ against *Ascosphaera apis*.

The results in Table 2 show that ozonide **1** and tetraoxane **6** were more potent than Triadimefon **9** against *A. apis.*, but inferior to Kresoxim-methyl **10**. Tetraoxane **3** exhibited activity similar to Triadimefon **9**. Despite the fact that IC_50_ of Kresoxim-methyl was lower than that of peroxides, Kresoxim-methyl did not completely inhibit the growth of mycelium of *A. apis* even at a concentration of 30 mg/L.

**Figure 3:**
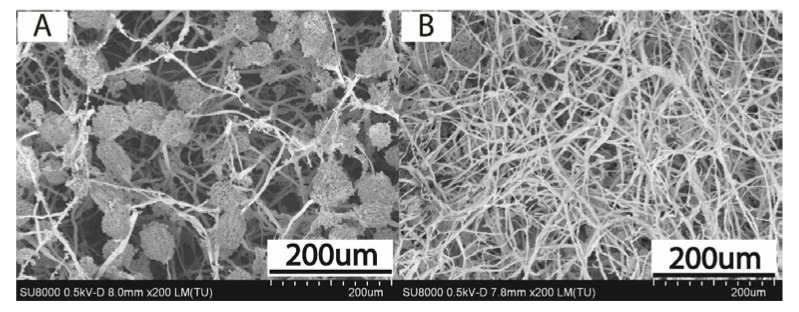
The characterization of *A. apis* by SEM image. (**A**) The control culture, (**B**) The culture treated with 3 mg/L of peroxide **6**.

In addition, SEM characterization of the control culture of *A. apis* and the mycelium of *A. apis* after 14 days incubation with peroxide **6** at 3 mg/L was carried out (Figure 3). The control culture contained globose sporocysts on the mycelium. *A. apis* treated with 3 mg/L of peroxide **6** almost did not contain sporocysts. The findings showed that peroxides did not give a chance to the fungus for its growth.

### 2.3. Evaluation of Toxicity of Peroxides towards Bumblebees

**Figure 4:**
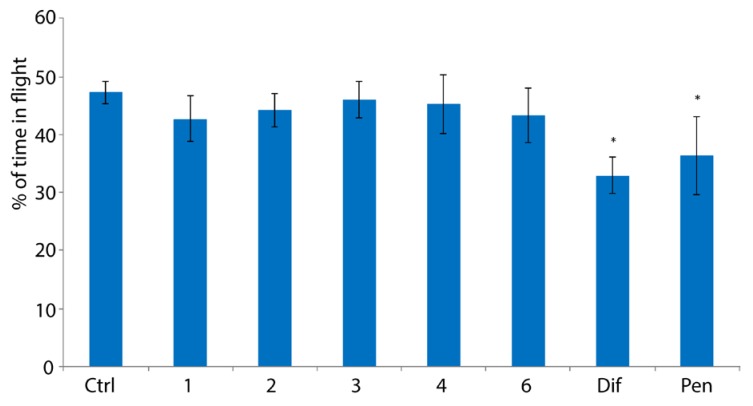
The effect of studied peroxides **1**–**4**, **6** and commercial fungicides (difenoconazole, penconazole) on the ability of bumblebees to fly after consuming sugar syrup with the test compound. * *p* < 0.005 Dif—difenoconazole, Pen—penconazole.

In the next step, we evaluated the toxicity of active peroxides **1**–**4**, **6** towards bumblebees. Two approaches were used for this purpose. The first approach was a contact action of bumblebees with 30 mg/L solution of peroxide in water containing DMSO (5% vol.). The second approach was feeding of bumblebees with inverted sugar syrup containing peroxides **1**–**4**, **6** (the concentration of peroxide was 30 mg/L) and DMSO (5% vol.). The mortality of bumblebees after their contact with solutions of peroxides **1**–**4**, **6** was absent. After consuming sugar syrup containing peroxide at a concentration of 30 mg/L, their mortality was not observed, and the ability to fly did not decrease. Moreover, in both approaches, all bumblebees were alive, even with an increase of the concentration of peroxides **1**–**4**, **6** by 10 times to 300 mg/L. In the case of commercial fungicides—difenoconazole and penconazole, they did not affect the ability to fly in the contact action test. Bumblebee mortality had not increased both in contact with difenoconazole and penconazole and consumption of syrup with these fungicides. However, after the consumption of sugar syrup containing difenoconazole or penconazole, the ability of bumblebees to fly was decreased by 1.4 times and 1.3 times, respectively (Figure 4). On average, bumblebees from the control group were in flight for 9 min 28 s. After the consumption of syrup with difenoconazole and penconazole (see Materials and methods), the time spent by insects in flight decreased to 6 min 36 s and 7 min 17 s, respectively.

## 3. Discussion

It was found that the synthetic cyclic peroxides possess fungicidal activity against pathogenic for bumblebees and honeybees microorganism—*Ascosphaera apis*. Ozonide **1** and tetraoxane **6** exhibited the strongest inhibitory effect on the growth of *A. apis* (EC_50_ = 4.0 mg/L, 1.6 mg/L, respectively). Peroxides **1**–**3** and **6** inhibited the growth of the mycelium of the fungus at range 94–100%. It is noteworthy that the classic fungicides—Kresoxim-methyl **10** and Triadimefon **9**—at a concentration of 30 mg/L did not suppress the growth of mycelium of *A. apis* by 100%.

Cyclic peroxides **1**–**4**, **6** did not reduce the ability of bumblebees to fly both after contact with 30 mg/L solution of peroxide in water containing DMSO (5% vol.) and feeding with peroxides in an inverted sugar syrup with 5% vol. DMSO. Peroxides **1**–**4**, **6** did not lead to the death of bumblebees both after their contact action with solutions of peroxides and after consumption of sugar syrup containing peroxides even at a concentration of peroxide of 300 mg/L. So, using these compounds for the treatment of ascopherosis in bumblebees will not reduce the pollination activity of insects. Contrarily, commercial fungicides–difenoconazole and penconazole—led to a decrease in the ability of bumblebees to fly after oral treatment.

It was disclosed that cyclic synthetic peroxides effectively inhibited the growth of pathogen of honeybees and bumblebees—*A. apis*. Considering not long-living structures of the studied peroxides, most likely, they will not lead to environmental pollution and can be used to safely treat insects.

The antifungal mechanism action of cyclic peroxides, unlike H_2_O_2_ and hydroperoxides, probably does not depend on their oxidative potential. In the previous studies, we did not observe a direct correlation between cyclic peroxide oxidative potential and cytotoxic effect in relation to the human liver, lung, prostate cancer, and normal cells. It has been demonstrated that the activity depends on the stereochemistry of chiral centers in cyclic peroxides [14,15,16]. The antimalarial effect of Artemisinin and related peroxides is connected with a homolytic decomposition of O–O bond with the formation of *O*-centered radicals and their further transformation into *C*-centered radicals, the action of which determines antimalarial activity. A similar mechanism of the antifungal action of peroxides can be supposed in our case.

## 4. Materials and Methods

### 4.1. Samples

The objects of the study were adult male bumblebees *Bombus terrestris* L. (more than 3 days after hatching from pupae), as well as bumblebee larvae provided by the Technology of Bumblebee Rearing Ltd. (Voronezh, Russia). Ozonide **1**, bridged 1,2,4,5-tetraoxanes **2**–**6** [25], and tricyclic monoperoxides **7**,**8** [55] were synthesized in accordance with previously reported procedures. Physical data and description of ^1^H and ^13^C-NMR spectra of peroxides **1**–**8** see in the Supplementary Materials.

### 4.2. Molecular Identification of Microorganisms in the Intestinal Contents of Bumblebee Larvae

The intestinal contents of bumblebee larvae were seeded on Saburo №2GRM medium (Russia): pancreatic hydrolysate of fish meal—10 g/L; pancreatic hydrolysate of casein—10 g/L; yeast extract—2 g/L; NaH_2_PO_4_—2 g/L; d-glucose—40 g/L; microbiological agar—10 g/L; pH 6.0. To suppress the growth of non-target microflora in the medium, 10 mL of 1% chloramphenicol solution per 1 L of the medium was added. DNA from the grown colonies on Saburo medium was isolated using commercially available PROBA-GS kit (DNA Technology, Russia) in accordance with recommendations of the manufacturer (https://www.dna-technology.ru/files/images/instruction/en-(110–2)PROBA-GS19.10.10.pdf). The polymerase chain reaction was performed with an Eppendorf MasterCycler Personal cycler. Each PCR reaction mixture in 0.25 mL test tube contained 5 µL of 5X reaction buffer (Evrogen, Moscow, Russia), 1 µL of 5 µM forward primer, 1 µL of 5 µM reverse primer, 2 µL of template DNA, and deionized water (up to 25 µL). PCR regime included initial denaturation at 94 °C for 3 min, 35 cycles of denaturation at 94 °C for 30 s, annealing at 54 °C for 30 s, elongation at 72 °C for 45 s, final elongation at 72 °C for 10 min. The following primers were used: forward ITS1 TCCGTAGGTGAACCTGCGG, reverse ITS4 TCCTCCGCTTATTGATATGC (White et al. 1990). PCR products were stained with ethidium bromide and visualized at 312 nm with TCO-20LM transilluminator after electrophoresis on 2% agarose gel. RCR products were extracted from the gel and purified with a commercially available Cleanup Standard kit (Evrogen, Russia) and sequenced with Applied Biosystems 3500 automated sequencer using BigDye Terminator v3.1 Cycle Sequencing Kit (Thermo Fisher Scientific, Waltham, MA, USA) and ITS1/ITS4 primers (Evrogen, Moscow, Russia). The following eukaryotic microorganisms were detected: *Lachancea thermotolerans*, *Mucor racemosus*, *Naganishia adeliensis*, *Penicillium commune*, *Rhodotorula mucilaginosa*, *Ascosphaera apis*.

### 4.3. Study of the Toxic Effect of the Peroxides on Bumblebees (Contact Action)

Peroxide **1**–**4**, **6** (0.3 mg or 3.0 mg) was added to 500 µL of DMSO, and the resulting solution was diluted with distilled water to 10 mL. After that, a 30 mg/L or 300 mg/L solution of peroxide in water containing DMSO (5% vol.) was obtained. A solution of DMSO (500 µL) in distilled water (9.5 mL) was used as the control solution. Bumblebees, gently with tweezers for 1 s, were placed in a test tube with the tested solution of peroxide **1**–**4**, **6** in water containing DMSO (5% vol.). After that, they were kept for 2 h in a specialized cage with filter paper at the bottom to dry. Then, bumblebees were placed in cylindrical cages (diameter-14 cm, height-7 cm) with a mesh bottom and a lid; 10 bumblebees in each cage. Inverted sugar syrup (60%) was used as a feed. Bumblebees were kept at a temperature of 27–28.5 °C and at a humidity of 55–68%. The number of dead bumblebees was registered after 3 days.

### 4.4. Study of the Toxic Effect of the Peroxides on Bumblebees (Feeding of Bumblebees)

Peroxide **1**–**4**, **6** (0.3 mg or 3.0 mg) was added to 500 µL of DMSO, and the resulting solution was diluted with 60% inverted sugar syrup to 10 mL. After that, 30 mg/L or 300 mg/L solution of peroxide in inverted sugar syrup containing DMSO (5% vol.) was obtained. A solution of DMSO (500 µL) in 60% inverted sugar syrup was used as the control solution. Bumblebees were placed in cylindrical cages (diameter-14 cm, height-7 cm) with a mesh bottom and a lid; 10 bumblebees in each cage. Bumblebees were kept at a temperature of 27–28.5 °C and at a humidity of 55–68%. The number of dead bumblebees was registered after 3 days.

### 4.5. Study of the Ability of Bumblebees to Fly

Bumblebees, which either were in contact with the solution of the studied compounds (see above Section 4.3) or the consumed syrup with the studied compounds (see above Section 4.4), were used. Bumblebees (3 insects) were placed in a transparent chamber: length 25 cm, width 15 cm, height 20 cm. For lighting, fluorescent lamp 600 mm 18W D26 G13 neutral-white PHILIPS was used. Further, the number of bumblebees in the state of the flight was registered every 5 s for 20 min (the optimal time period required for measuring the ability of bumblebees to fly). During this time, the lamp was turned on. After that, the average number of bumblebees that were in flight was calculated. 

### 4.6. Evaluation of Fungicidal Activity of the Peroxides

The fungicidal activity was tested according to the common procedure with *Ascosphaera apis* [56]. Compounds were dissolved in acetone to prepare a 3 mg/mL stock solution. Then, aliquots of the stock solution were diluted with acetone to obtain a series of solutions with a concentration of 0.03–3 mg/mL. After that, the aliquots of the tested compound were mixed with potato-saccharose agar at 50 °C to obtain the 0.3–30 µg/mL concentration of the compound. The final acetone concentration of both fungicide-containing and control samples was 10 mL/L. Petri dishes containing 15 mL of the agar medium were inoculated by placing 2-mm mycelial agar discs on the agar surface. Plates were incubated at 25 °C for 72 h. A mixed medium without a sample was used as blank control. After incubation, the mycelium elongation diameter (mm) of fungi settlements was measured. Three replicates of each test were carried out. The growth inhibition rates were calculated with the following equation: I = [(DC − DT)/DC] × 100%. Here, I is the growth inhibition rates (%), DC is the control settlement diameter (mm), and DT is the treatment group fungi settlement diameter (mm). Commercially available agricultural fungicides—Triadimefon and Kresoxim-methyl—were used as positive controls. EC_50_ values were calculated by non-linear regression using an equation for a sigmoidal dose-response curve with variable slope (Prism 7.0, GraphPad Software, San Diego, CA, USA).

### 4.7. SEM Characterization of Ascosphaera apis

The target-oriented approach was utilized for the optimization of the analytic measurements [57]. Before measurements, the samples were mounted on a 25 mm aluminum specimen stub and fixed by conductive carbon paint. Metal coating with a thin film (10 nm) of gold/palladium alloy (60/40) was performed using a magnetron sputtering method, as described earlier [58]. The observations were carried out using a Hitachi SU8000 field-emission scanning electron microscope (FE-SEM, Hitachi, Tokyo, Japan). Images were acquired in deceleration mode at 500 V landing voltage. Signal summing (secondary electrons (SE) + backscattered electrons (BSE)) was employed during the observations.

## 5. Conclusions

Cyclic peroxides exhibited high antifungal activity against entomopathogenic fungus *Ascosphaera apis*. The globose sporocysts on the mycelium of *A. apis* were practically absent under the action of peroxides. The peroxides were non-toxic to bumblebees and did not reduce their ability to fly. Some peroxides were more effective against *A. apis* than commercial fungicide Triadimefon. Thus, cyclic peroxides could be considered as effective agents against the pathogen of bumblebees and honeybees—*A. apis*, which are at the same safe for pollinators.

## Figures and Tables

**Table 1:** Mycelium growth inhibition (I, %) of *A. apis* by peroxides **1**–**8**, Triadimefon **9**, and Kresoxim-methyl **10** at a concentration of 30 mg/L.

Compound	Mycelium Growth Inhibition (I), %
**1**	94
**2**	100
**3**	100
**4**	66
**5**	49
**6**	94
**7**	53
**8**	25
Triadimefon **9**	91
Kresoxim-methyl **10**	88

**Table 2:** Fungicidal activity (EC_50_) of peroxides (**1**–**3**, **6**) and the reference compounds.

Compound	1	2	3	6	Triadimefon, 9	Kresoxim-Methyl, 10
**EC_50_ (mg/L) ± SD**	4.0 ± 0.2	10.9 ± 0.7	7.5 ± 1.0	1.6 ± 0.1	7.1 ± 0.8	<1.0

## Supplementary Materials

The following are available online. Physical data and description of ^1^H and ^13^C-NMR spectra of peroxides **1**–**8**.

## References

[1] Fisher L.C., Blackie M.A. (2014). Tetraoxanes as antimalarials: Harnessing the endoperoxide. Mini-Rev. Med. Chem..

[2] Ghorai P., Dussault P.H., Hu C. (2008). Synthesis of spiro-bisperoxyketals. Org. Lett..

[3] Hao H.D., Wittlin S., Wu Y. (2013). Potent antimalarial 1,2,4-trioxanes through perhydrolysis of epoxides. Chem. Eur. J..

[4] Jefford C.W. (2012). Synthetic Peroxides as Potent Antimalarials. News and Views. Curr. Top. Med. Chem..

[5] Opsenica D.M., Šolaja B.A. (2009). Antimalarial peroxides. J. Serb. Chem. Soc..

[6] Šolaja B.A., Terzić N., Pocsfalvi G., Gerena L., Tinant B., Opsenica D., Milhous W.K., Fisher L.C., Blackie M.A. (2014). Mixed steroidal 1,2,4,5-tetraoxanes: Antimalarial and antimycobacterial activity. J. Med. Chem..

[7] Cowan N., Yaremenko I.A., Krylov I.B., Terent’ev A.O., Keiser J. (2015). Elucidation of the in vitro and in vivo activities of bridged 1,2,4-trioxolanes, bridged 1,2,4,5-tetraoxanes, tricyclic monoperoxides, silyl peroxides, and hydroxylamine derivatives against Schistosoma mansoni. Bioorgan. Med. Chem..

[8] Ingram K., Yaremenko I.A., Krylov I.B., Hofer L., Terentev A.O., Keiser J. (2012). Identification of antischistosomal leads by evaluating bridged 1,2,4,5-tetraoxanes, alphaperoxides, and tricyclic monoperoxides. J. Med. Chem..

[9] Keiser J., Ingram K., Vargas M., Chollet J., Wang X., Dong Y., Vennerstrom J.L. (2012). In vivo activity of aryl ozonides against Schistosoma species. Antimicrob. Agents Chemother..

[10] Küster T., Kriegel N., Stadelmann B., Wang X., Dong Y., Vennerstrom J.L., Keiser J., Hemphill A. (2014). Amino ozonides exhibit in vitro activity against Echinococcus multilocularis metacestodes. Int. J. Antimicrob. Agents.

[11] Vil’ V.A., Yaremenko I.A., Ilovaisky A.I., Terent’ev A.O. (2017). Peroxides with Anthelmintic, Antiprotozoal, Fungicidal and Antiviral Bioactivity: Properties, Synthesis and Reactions. Molecules.

[12] Abrams R.P., Carroll W.L., Woerpel K.A. (2016). Five-Membered Ring Peroxide Selectively Initiates Ferroptosis in Cancer Cells. ACS Chem. Biol..

[13] Chaudhari M.B., Moorthy S., Patil S., Bisht G.S., Mohamed H., Basu S., Gnanaprakasam B. (2018). Iron-Catalyzed Batch/Continuous Flow C-H Functionalization Module for the Synthesis of Anticancer Peroxides. J. Org. Chem..

[14] Yaremenko I.A., Syroeshkin M.A., Levitsky D.O., Fleury F., Terent’ev A.O. (2017). Cyclic peroxides as promising anticancer agents: In vitro cytotoxicity study of synthetic ozonides and tetraoxanes on human prostate cancer cell lines. Med. Chem. Res..

[15] Coghi P., Yaremenko I.A., Prommana P., Radulov P.S., Syroeshkin M.A., Wu Y.J., Gao J.Y., Gordillo F.M., Mok S., Wong V.K.W. (2018). Novel Peroxides as Promising Anticancer Agents with Unexpected Depressed Antimalarial Activity. Chemmedchem.

[16] Yaremenko I.A., Coghi P., Prommana P., Qiu C., Radulov P.S., Qu Y., Belyakova Y.Y., Zanforlin E., Kokorekin V.A., Wu Y.Y.J. (2020). Synthetic Peroxides Promote Apoptosis of Cancer Cells by Inhibiting P-Glycoprotein ABCB5. Chemmedchem.

[17] Dwivedi A., Mazumder A., du Plessis L., du Preez J.L., Haynes R.K., du Plessis J. (2015). In vitro anti-cancer effects of artemisone nano-vesicular formulations on melanoma cells. Nanomed.-Nanotechnol..

[18] Chou S.W., Marousek G., Auerochs S., Stamminger T., Milbradt J., Marschall M. (2011). The unique antiviral activity of artesunate is broadly effective against human cytomegaloviruses including therapy-resistant mutants. Antivir. Res.

[19] Efferth T., Romero M.R., Wolf D.G., Stamminger T., Marin J.J.G., Marschall M. (2008). The antiviral activities of artemisinin and artesunate. Clin. Infect. Dis..

[20] Reiter C., Frohlich T., Gruber L., Hutterer C., Marschall M., Voigtlander C., Friedrich O., Kappes B., Efferth T., Tsogoeva S.B. (2015). Highly potent artemisinin-derived dimers and trimers: Synthesis and evaluation of their antimalarial, antileukemia and antiviral activities. Bioorgan. Med. Chem..

[21] Chaudhary S., Sharma V., Jaiswal P.K., Gaikwad A.N., Sinha S.K., Puri S.K., Sharon A., Maulik P.R., Chaturvedi V. (2015). Stable Tricyclic Antitubercular Ozonides Derived from Artemisinin. Org. Lett..

[22] Miller M.J., Walz A.J., Zhu H., Wu C., Moraski G., Möllmann U., Tristani E.M., Crumbliss A.L., Ferdig M.T., Checkley L. (2011). Design, Synthesis, and Study of a Mycobactin−Artemisinin Conjugate That Has Selective and Potent Activity against Tuberculosis and Malaria. J. Am. Chem. Soc..

[23] Zhou F.W., Lei H.S., Fan L., Jiang L., Liu J., Peng X.M., Xu X.R., Chen L., Zhou C.H., Zou Y.Y. (2014). Design, synthesis, and biological evaluation of dihydroartemisinin- fluoroquinolone conjugates as a novel type of potential antitubercular agents. Bioorgan. Med. Chem. Lett..

[24] NobelPrize.org Tu Youyou—Facts. Nobel Media AB 2020. https://www.nobelprize.org/prizes/medicine/2015/tu/facts/.

[25] Yaremenko I.A., Radulov P.S., Belyakova Y.Y., Demina A.A., Fomenkov D.I., Barsukov D.V., Subbotina I.R., Fleury F., Terent’ev A. (2020). Catalyst development for the synthesis of ozonides and tetraoxanes under heterogeneous conditions. Disclosure of an unprecedented class of fungicides for agricultural application. Chem. Eur. J..

[26] Potts S.G., Biesmeijer J.C., Kremen C., Neumann P., Schweiger O., Kunin W.E. (2010). Global pollinator declines: Trends, impacts and drivers. Trends Ecol. Evol..

[27] Biesmeijer J.C., Roberts S.P.M., Reemer M., Ohlemuller R., Edwards M., Peeters T., Schaffers A.P., Potts S.G., Kleukers R., Thomas C.D. (2006). Parallel declines in pollinators and insect-pollinated plants in Britain and the Netherlands. Science.

[28] Thomann M., Imbert E., Devaux C., Cheptou P.O. (2013). Flowering plants under global pollinator decline. Trends Plant Sci..

[29] Rhodes C.J. (2018). Pollinator decline-an ecological calamity in the making?. Sci. Prog..

[30] Klein A.M., Vaissiere B.E., Cane J.H., Steffan-Dewenter I., Cunningham S.A., Kremen C., Tscharntke T. (2007). Importance of pollinators in changing landscapes for world crops. Proc. Roy. Soc. B.

[31] Simon-Delso N., Amaral-Rogers V., Belzunces L.P., Bonmatin J.M., Chagnon M., Downs C., Furlan L., Gibbons D.W., Giorio C., Girolami V. (2015). Systemic insecticides (neonicotinoids and fipronil): Trends, uses, mode of action and metabolites. Environ. Sci. Pollut. R..

[32] Bryden J., Gill R.J., Mitton R.A.A., Raine N.E., Jansen V.A.A. (2013). Chronic sublethal stress causes bee colony failure. Ecol. Lett..

[33] Tosi S., Nieh J.C. (2017). A common neonicotinoid pesticide, thiamethoxam, alters honey bee activity, motor functions, and movement to light. Sci. Rep..

[34] Tosi S., Burgio G., Nieh J.C. (2017). A common neonicotinoid pesticide, thiamethoxam, impairs honey bee flight ability. Sci. Rep..

[35] Wu-Smart J., Spivak M. (2018). Effects of neonicotinoid imidacloprid exposure on bumble bee (Hymenoptera: Apidae) queen survival and nest initiation. Environ. Entomol..

[36] Tosi S., Nieh J.C., Sgolastra F., Cabbri R., Medrzycki P. (2017). Neonicotinoid pesticides and nutritional stress synergistically reduce survival in honey bees. Proc. Roy. Soc. B.

[37] Fisher A., Coleman C., Hoffmann C., Fritz B., Rangel J. (2017). The Synergistic Effects of Almond Protection Fungicides on Honey Bee (Hymenoptera: Apidae) Forager Survival. J. Econ. Entomol..

[38] Raimets R., Karise R., Mand M., Kaart T., Ponting S., Song J.M., Cresswell J.E. (2018). Synergistic interactions between a variety of insecticides and an ergosterol biosynthesis inhibitor fungicide in dietary exposures of bumble bees (*Bombus terrestris* L.). Pest Manag. Sci..

[39] Zhu Y.C., Yao J.X., Adamczyk J., Luttrell R. (2017). Feeding toxicity and impact of imidacloprid formulation and mixtures with six representative pesticides at residue concentrations on honey bee physiology (Apis mellifera). PLoS ONE.

[40] Sgolastra F., Medrzycki P., Bortolotti L., Renzi M.T., Tosi S., Bogo G., Teper D., Porrini C., Molowny-Horas R., Bosch J. (2017). Synergistic mortality between a neonicotinoid insecticide and an ergosterol-biosynthesis-inhibiting fungicide in three bee species. Pest Manag. Sci..

[41] DeGrandi-Hoffman G., Corby-Harris V., DeJong E.W., Chambers M., Hidalgo G. (2017). Honey bee gut microbial communities are robust to the fungicide PristineA (R) consumed in pollen. Apidologie.

[42] Steffan S.A., Dharampal P.S., Diaz-Garcia L., Currie C.R., Zalapa J., Hittinger C.T. (2017). Empirical, Metagenomic, and Computational Techniques Illuminate the Mechanisms by which Fungicides Compromise Bee Health. J. Vis. Exp..

[43] Kakumanu M.L., Reeves A.M., Anderson T.D., Rodrigues R.R., Williams M.A. (2016). Honey Bee Gut Microbiome Is Altered by In-Hive Pesticide Exposures. Front. Microbiol..

[44] Syromyatnikov M.Y., Kokina A.V., Lopatin A.V., Starkov A.A., Popov V.N. (2017). Evaluation of the toxicity of fungicides to flight muscle mitochondria of bumblebee (*Bombus terrestris* L.). Pestic. Biochem. Phys..

[45] Lopez J.H., Krainer S., Engert A., Schuehly W., Riessberger-Galle U., Crailsheim K. (2017). Sublethal pesticide doses negatively affect survival and the cellular responses in American foulbrood-infected honeybee larvae. Sci. Rep..

[46] Grassl J., Holt S., Cremen N., Peso M., Hahne D., Baer B. (2018). Synergistic effects of pathogen and pesticide exposure on honey bee (Apis mellifera) survival and immunity. J. Invertebr. Pathol..

[47] O’Neill P.M., Amewu R.K., Charman S.A., Sabbani S., Gnadig N.F., Straimer J., Fidock D.A., Shore E.R., Roberts N.L., Wong M.H.L. (2017). A tetraoxane-based antimalarial drug candidate that overcomes PfK13-C580Y dependent artemisinin resistance. Nat. Commun..

[48] Aufauvre J., Biron D.G., Vidau C., Fontbonne R., Roudel M., Diogon M., Vigues B., Belzunces L.P., Delbac F., Blot N. (2012). Parasite-insecticide interactions: A case study of Nosema ceranae and fipronil synergy on honeybee. Sci. Rep..

[49] Spiltoir C.F. (1955). Life cycle of Ascosphaera Apis (Pericystis Apis). Am. J. Bot..

[50] Zaghloul O., Mourad A.K., EI Kady M.B., Nemat F., Morsy M.E. (2005). Assessment of losses in honey yield due to the chalkbrood disease, with reference to the determination of its economic injury levels in Egypt. Commun. Agric. Appl. Biol. Sci..

[51] Bailey L., Ball B.V., Bailey L., Ball B.V. (1991). The Treatment of Bee Diseases. Honey Bee Pathology.

[52] Přidal A., Sedláček I., Marvanová L. (1997). Microbiology of Bombus terrestris L. larvae (Hymenoptera:Apoidea) from laboratory rearing. Acta Univ. Agric. Silvic. Mendel Brun..

[53] Maxfield-Taylor S.A., Mujic A.B., Rao S. (2015). First Detection of the Larval Chalkbrood Disease Pathogen Ascosphaera apis (Ascomycota: Eurotiomycetes: Ascosphaerales) in Adult Bumble Bees. PLoS ONE.

[54] Pereira K.D., Meeus I., Smagghe G. (2019). Honey bee-collected pollen is a potential source of Ascosphaera apis infection in managed bumble bees. Sci. Rep..

[55] Terent’ev A.O., Yaremenko I.A., Chernyshev V.V., Dembitsky V.M., Nikishin G.I. (2012). Selective Synthesis of Cyclic Peroxides from Triketones and H2O2. J. Org. Chem..

[56] (1984). Metodicheskie Rekomendatsii po Opredeleniyu Fungitsidnoi Aktivnosti Novykh Soedinenii [Guidelines for Determination of Fungicidal Activity of New Compounds].

[57] Kachala V.V., Khemchyan L.L., Kashin A.S., Orlov N.V., Grachev A.A., Zalesskiy S.S., Ananikov V.P. (2013). Target-oriented analysis of gaseous, liquid and solid chemical systems by mass spectrometry, nuclear magnetic resonance spectroscopy and electron microscopy. Russ. Chem. Rev..

[58] Kashin A.S., Ananikov V.P. (2011). A SEM study of nanosized metal films and metal nanoparticles obtained by magnetron sputtering. Russ. Chem. Bull. Int. Ed..

