# Supplementary material for: Cyclic Synthetic Peroxides Inhibit Growth of Entomopathogenic Fungus Ascosphaera apis without Toxic Effect on Bumblebees
Source: Molecules. 2020 Apr 22;25(8):1954. doi: 10.3390/molecules25081954 (PMC7221740; doi:10.3390/molecules25081954)
Supplement: Supplementary file 1 [file molecules-25-01954-s001.zip › Supporting Information/Supporting information.doc]

**Supporting information**

Cyclic synthetic peroxides inhibit growth of entomopathogenic fungus *Ascosphaera apis* without toxic effect on bumblebees

Ivan A. Yaremenko^1,2^, Mikhail Y. Syromyatnikov^3^, Peter S. Radulov ^1,2^, Yulia Yu. Belyakova ^1,2^, Dmitriy I. Fomenkov ^1^, Vasily N. Popov^3,4^, and Alexander O. Terent’ev ^1,2^*

^1^	N. D. Zelinsky Institute of Organic Chemistry, Russian Academy of Sciences, Moscow, 119991, Russia

^2^	All-Russian Research Institute for Phytopathology, B. Vyazyomy, Moscow Region, 143050, Russia

^3^	Department of Genetics, Cytology and Bioengineering, Voronezh State University, Voronezh, 394018, Russia

^4^ Voronezh State University of Engineering Technologies, Voronezh, 394036, Russia.

*****	Correspondence: [alterex@yandex.ru](mailto:alterex@yandex.ru) Tel.: +7-916-385-4080

**Table of contents**

1. Physical data of peroxides 1-8	2

2. The nucleotide sequences of detected eukaryotic microorganisms	3

# 1. Physical data of peroxides 1-8

***Ethyl-2-allyl-1,5-dimethyl-6,7,8-trioxabicyclo[3.2.1]octane-2-carboxylate (1)***

The product was obtained as a slightly yellow oil in the yield of 75% (0.240 g). ^1^Н NMR (300.13 MHz, CDCl_3_), *δ*: 1.22–1.31 (m, 6H), 1.47 (s, 3H), 1.50 (s, 3H), 1.57 (s, 3H), 1.68 (s, 3H), 1.61-1.83 (m, 3H), 1.87-2.22 (m, 5H), 2.42-2.52 (m, 1H), 2.58-2.75 (3H), 4.11-4.23 (m, 4H), 5.01-5.13 (m, 4H), 5.52-5.72 (m, 2H); ^13^С NMR (75.48 MHz, CDCl_3_), *δ*: 14.3, 18.7, 18.8, 20.7, 20.8, 22.7, 26.0, 30.8, 32.9, 36.4 39.8, 52.8, 53.0, 61.1, 61.2, 109.0, 109.8, 110.9, 118.7, 118.9, 132.4, 133.7, 172.4, 172.6. Anal. Calcd. for C_13_H_20_O_5_: C, 60.92; H, 7.87. Found: C, 60.85; H, 7.79. The physical and spectral data were consistent with those previously reported.

***7-Allyl-1,4-dimethyl-2,3,5,6-tetraoxabicyclo[2.2.1]heptane (2)***

The product was obtained as a slightly yellow oil in the yield of 55% (0.203 g). ^1^H NMR (300.13 MHz, CDCl_3_) *δ*: 1.53 (s, 6H), 2.38 (t, *J* = 6.7 Hz, 2H), 2.78 (t, *J* = 6.7 Hz, 1H), 5.15–5.20 (m, 2H), 5.80–5.93 (m, 1H). ^13^C NMR (75.48 MHz, CDCl_3_) *δ*: 10.0, 28.6, 57.9, 110.7, 118.0, 134.1. Anal. Calcd. for C_8_H_12_O_4_: C, 55.81; H, 7.03. Found: C, 55.72; H, 6.98. The physical and spectral data were consistent with those previously reported.

***1,4-Dimethyl-7-pentyl-2,3,5,6-tetraoxabicyclo[2.2.1]heptane (3)***

The product was obtained as a slightly yellow oil in the yield of 70% (0.250 g). ^1^H NMR (300.13 MHz, CDCl_3_) *δ*: 0.91 (t, *J* = 7.0 Hz, 3H), 1.34–1.37 (m, 4H), 1.46 – 1.48 (m, 2H), 1.54 (s, 6H), 1.55–1.62 (m, 2H), 2.62 (t, *J* = 5.8 Hz, 1H). ^13^C NMR (75.48 MHz, CDCl_3_) *δ*: 10.0, 14.1, 22.5, 24.0, 27.5, 32.1, 59.3, 111.0. Anal. Calcd. for C_10_H_18_O_4_: C, 59.39; H, 8.97. Found: C, 59.43; H, 8.85. The physical and spectral data were consistent with those previously reported.

***7-Isopentyl-1,4-dimethyl-2,3,5,6-tetraoxabicyclo[2.2.1]heptane (4)***

The product was obtained as a slightly yellow oil in the yield of 64% (0.228 g). ^1^H NMR (300.13 MHz, CDCl_3_) *δ*: 0.93 (d, *J* = 6.6 Hz, 6H), 1.30–1.40 (m, 2H), 1.54 (s, 6H), 1.50-1.64 (m, 3H) 2.59 (t, *J* = 6.6 Hz, 1H). ^13^C NMR (75.48 MHz, CDCl_3_) *δ*: 10.0, 21.9, 22.5, 28.4, 36.9, 59.5, 110.9. Anal. Calcd. for C_10_H_18_O_4_: C, 59.39; H, 8.97. Found: C, 59.31; H, 8.90. The physical and spectral data were consistent with those previously reported.

***7-Hexyl-1,4-dimethyl-2,3,5,6-tetraoxabicyclo[2.2.1]heptane (5)***

The product was obtained as a slightly yellow oil in the yield of 60% (0.211 g). ^1^H NMR (300.13 MHz, CDCl_3_) *δ*: 0.92 (t, *J* = 7.0 Hz, 3H), 1.27 – 1.41 (m, 6H), 1.43–1.54 (m, 2H), 1.56 (s, 6H), 1.58–1.54 (m, 2H), 2.64 (t, *J* = 5.9 Hz, 1H). ^13^C NMR (75.48 MHz, CDCl_3_) *δ*: 10.0, 14.2, 22.7, 24.0, 27.8, 29.6, 31.7, 59.3, 111.0. Anal. Calcd. for C_11_H_20_O_4_: C, 61.09; H, 9.32. Found: C, 59.92; H, 9.22. The physical and spectral data were consistent with those previously reported.

***7-(Adamantan-1-yl)-1,4-dimethyl-2,3,5,6-tetraoxabicyclo[2.2.1]heptane (6)***

The product was obtained as a white crystals in the yield of 85% (0.289 g), Mp = 130–131°C (Lit. Mp = 131–132 °C). ^1^H NMR (300.13 MHz, CDCl_3_) *δ*: 1.68-2.04 (m, 21H), 2.40 (s, 1H). ^13^C NMR (75.48 MHz, CDCl_3_) *δ*: 12.8, 28.5, 33.1, 36.9, 40.8, 67.0, 110.7. Anal. Calcd. for C_15_H_22_O_4_: C, 67.65; H, 8.33. Found: C, 67.58; H, 8.21. The physical and spectral data were consistent with those previously reported.

***3-(4-Methoxyphenyl)-6,7a-dimethyltetrahydro-3H,4H-3,6-epoxy[1,2]dioxolo[3,4-b]pyran (7)***

The product was obtained as a white crystals in the yield of 38% (0.120 g), Mp = 89–90°C (Lit. Mp = 89–90°C). ^1^H NMR (300.13 MHz, CDCl_3_): δ 1.55 (s, 3H), 1.61 (s, 3H), 1.68-1.80 (m, 4H), 2.62-2.66 (m, H), 3.80 (s, 3H), 6.90 (d, 2H, *J* = 8.8 Hz), 7.49 (d, 2H, *J* = 8.8 Hz). ^13^C NMR (75.48 MHz, CDCl_3_): δ 12.5, 17.9, 24.8, 29.3, 50.5, 55.3, 95.8, 105.6, 106.5, 113.9, 124.7, 128.1, 160.5. Anal. Calcd. for C_15_H_18_O_5_: C, 64.74; H, 6.52. Found: C, 64.70; H, 6.61. The physical and spectral data were consistent with those previously reported.

***3a-Allyl-3,6,7a-trimethyltetrahydro-3H,4H-3,6-epoxy[1,2]-dioxolo[3,4-b]pyran (8)***

The product was obtained as a white crystals in the yield of 63% (0.203 g), Mp = 43–44°C (Lit. Mp = 43–44°C). ^1^H NMR (300.13 MHz, CDCl_3_): δ 1.39 (s, 3H), 1.47 (s, 6H), 1.70-1.72 (m, 4H), 2.27 (d, 2H, *J* = 7.3 Hz), 5.04-5.15 (m, 2H), 5-79-5.95 (m, H). ^13^C NMR (75.48 MHz, CDCl3): δ 16.1, 19.0, 24.7, 30.8, 35.8, 50.7, 94.0, 107.1, 118.5, 133.2. Anal. Calcd. for C_12_H_18_O_4_: C, 63.70; H, 8.02. Found: C, 63.65; H, 7.90. The physical and spectral data were consistent with those previously reported.

1.	Yaremenko, I.A.; Radulov, P.S.; Belyakova, Y.Y.; Demina, A.A.; Fomenkov, D.I.; Barsukov, D.V.; Subbotina, I.R.; Fleury, F.; Terent'ev, A. Catalyst development for the synthesis of ozonides and tetraoxanes under heterogeneous conditions. Disclosure of an unprecedented class of fungicides for agricultural application. *Chem. Eur. J.* **2019**, DOI:10.1002/chem.201904555.

2.	Terent'ev, A.O.; Yaremenko, I.A.; Chernyshev, V.V.; Dembitsky, V.M.; Nikishin, G.I. Selective Synthesis of Cyclic Peroxides from Triketones and H2O2. *J. Org. Chem.* **2012,** *77*, (4), 1833-1842.

# 2. The nucleotide sequences of detected eukaryotic microorganisms

>1. *Ascosphaera apis*

GACAAAGGTGGAGTGCGCGGCAGGCCTTGTTTAGGGGCACCTAGCCGCACAGACACACATTAATGATCCCTTCCGTAGGGGTGACCTGCGGAAGGATCATTAATGTGTGTCTGTGCGGCTAGGTGCCCCTAAACAAGGCCCTGCCGCGCACTCCCACCCTTGTCTACCTTACCTGTTGCTTCGGCGGGCCTGCGGGTTCTCGCGAGCCTGCTGCCGGAGGGGTTAGTTCCCCCCTGGCTAGCGTCCGCCGAAGATAAACGAACTCCAGTCGAAGATTGAAGTCTGAAGAAAATTGATAAATAAATCAAAACTTTCAACAACGGATCTCTTGGTTCCGACATCGATGAAGAACGCAGCGAAATGCGATAAGTAATGTGAATTGCAGAATTCCGTGAATCATCGAATCTTTGAACGCACATTGCGCCCTCTGGTATTCCGGGGGGCATGCCTGTCCGAGCGTCATTGCAACCCTCAAGCACGGCTTGTGTGTTGGGCGATCGTCCCGTCTTAGGAGGGACGCGCCCGAAAGGCAGTGACGGCGTCGTGTTCCGGTGCCCGAGCGTATGGGGCTTTGTCTTTCGCTCTAGTGGCCTGGCCGACTGTCCGGTCTAACCATCATTTACTTCTAGTGGTTGACCTCGGATCAGGTAGGGTTACCCGCTGAACTTAAGCATATCAATAAGCGGGAGGAA

>2. *Penicillium commune*

GCGTGAGCCCCCGGCGGCCAGTTAGGCGGCCCGCCGAAGCAACAAGGTAAAATAAAACACGGTGGAGTTGGACCAGAGGGCCCTCACTCGGCTTCTGTCGGGTGAACCTGCGGAAGGATCATTACCGAGTGAGGGCCCTCTGGGTCCAACCTCCCACCCGTGTTTATTTTACCTTGTTGCTTCGGCGGGCCCGCCTTAACTGGCCGCCGGGGGGCTCACGCCCCCGGGCCCGCGCCCGCCGAAGACACCCTCGAACTCTGTCTGAAGATTGAAGTCTGAGTGAAAATATAAATTATTTAAAACTTTCAACAACGGATCTCTTGGTTCCGGCATCGATGAAGAACGCAGCGAAATGCGATACGTAATGTGAATTGCAAATTCAGTGAATCATCGAGTCTTTGAACGCACATTGCGCCCCCTGGTATTCCGGGGGGCATGCCTGTCCGAGCGTCATTGCTGCCCTCAAGCCCGGCTTGTGTGTTGGGCCCCGTCCTCCGATTTCCGGGGGACGGGCCCGAAAGGCAGCGGCGGCACCGCGTCCGGTCCTCGAGCGTATGGGGCTTTGTCACCCGCTCTGTAGGCCCGGCCGGCGCTTGCCGATCAACCCAAATTTTTTATCCAGGTTGACCTCGGATCAGGTAGGGATACCCGCTGAACTTAAGCATATCAATAAAGCGATAGATTACCGAGTGAGGGCCCTCTGGGTCAACCTCCCACCCGTGTTTTATTTTACCTTGTTGCTTCGGCGGGGCCGCCTTAACTGGGCCGCCGGGGGGCTCACGCCCCGGGCC

>3. *Lachancea thermotolerans*

TTTCCGTAGGGTGAACCTGCGGAAGGATCATTAAAGAATTTTGTTAGAGCAGCCGGGAAAGTTCAGAAGCCTGCGCTTGATTGCGCGGCCGATGATGCTTTCTGTTAACGACTGTCTCTCTACACACACACTGTGGAGTAATTTATTTTACAACGCTTCTTCTTTGGGCTTTACGGCCCAAGGGTTACAAACACAAACAACTATTGTATTTTAAACATTGTCAATTATTTTTCATTTTAGAAAAAAAATATTTAAAACTTTCAACAACGGATCTCTTGGTTCTCGCATCGATGAAGAACGCAGCGAAATGCGATAAGTATTGTGAATTGCAGATATTCGTGAATCATCGAATCTTTGAACGCACATTGCGCCCTCTGGTATTCCAGGGGGCATGCCTGTTTGAGCGTCATTTCCTTCTCAAACCCTCGGGTTTGGTAGTGAGTGGTACTCTTTCTGGGTTAACTTGAAAATGCTGGCCATCTGGCTGTTGCTGACTGAGGTTTTAGTCCAGTCCGCTGATACTCTGCGTATTAGGTTTTACCAACTCGTAGTGGCGTTAGTAGGCGTTTTAAAGGCTTTTACTGAAAGTACAGACAGTCTGGCAAACAGTATTCATAAAGTTTGACCTCAAATCAGGTAGGATTACCCGCTGAACTTAAGCATATCAATAAGCCG

>4. *Rhodotorula mucilaginosa*

TTCCGTAGGGTGAACCTGCGGAAGGATCATTAGTGAATATAGGACGTCCAACTTAACTTGGAGTCCGAACTCTCACTTTCTAACCCTGTGCACTTGTTTGGGATAGTAACTCTCGCAAGAGAGCGAACTCCTATTCACTTATAAACACAAAGTCTATGAATGTATTAAATTTTATAACAAAATAAAACTTTCAACAACGGATCTCTTGGCTCTCGCATCGATGAAGAACGCAGCGAAATGCGATAAGTAATGTGAATTGCAGAATTCAGTGAATCATCGAATCTTTGAACGCACCTTGCGCTCCATGGTATTCCGTGGAGCATGCCTGTTTGAGTGTCATGAATACTTCAACCCTCCTCTTTCTTAATGATTGAAGAGGTGTTTGGTTTCTGAGCGCTGCTGGCCTTTACGGTCTAGCTCGTTCGTAATGCATTAGCATCCGCAATCGAACTTCGGATTGACTTGGCGTAATAGACTATTCGCTGAGGAATTCTAGTCTTCGGATTAGAGCCGGGTTGGGTTAAAGGAAGCTTCTAATCAGAATGTCTACATTTTAAGATTAGATCTCAAATCAGGTAGGACTACCCGCTGAACTTAAGCATATCAATAAGCGGAGGAAAGGATCATTAGTGAATATAGGACGTCCAACTTAACTTGGAGTCCGAACTCTCACTTTCTACCCTGTGCACTTGTTTGGGATAGTAACTCT

>5. *Mucor racemosus*

TTCCGTAGGTGAACCTGCGGAAGGATCATTAAATAATCAATAATCTTGGCTTGTCCATTATTATCTATTTACTGTGAACTGTATTATTATTTGACATTTGAGGGATGTTCCAATGTTATAAGGATAGACATTGGAAATGTTAACCGAGTCATAATCAGGTTTAGGCCTGGTATCCTATTATTATTTACCAAATGAATTCAGAATTAATATTGTAACATAGACCTAAAAAATCTATAAAACAACTTTTAACAACGGATCTCTTGGTTCTCGCATCGATGAAGAACGTAGCAAAGTGCGATAACTAGTGTGAATTGCATATTCAGTGAATCATCGAGTCTTTGAACGCAACTTGCGCTCATTGGTATTCCAATGAGCACGCCTGTTTCAGTATCAAAACAAACCCTCTATCCAACTTTTGTTGTATAGGATTATTGGGGGCCTCTCGATCTGTATAGATCTTGAAACCCCTGAAATTTACTAAGGCCTGAACTTGTTTAAATGCCTGAACTTTTTTTTAATATAAAGGAAAGCTCTTGTAATTGACTTTGATGGGGCCTCCCAAATAAATCTTTTTTAAATTTGATCTGAAATCAGGCGGGATTACCCGCTGAACTTAAGCATATCAATAAGCGGAGGAAAGAGAGATCATTAAAATAATCAATAATCTTGGCTTGTCCATTATTATCTATTTACTGTGAACTGTATTATTATTTGACATTTGAGGGATGTTCCAATGTTATAAAGATAGACATTGGAAATGTTAACCGAGTCATAATCAGTTTAGCCTGGTATCCTATTATTATTTACCAAATGAATTC

>6. *Naganishia adeliensis*

GATCCAACCCTGCGTCTTCACTAAGCCCTCCGACAAGTGCACAGGTGTTAGGATTGATGTGCCTGTGAGCAAGCTCGACAGACGTTCAATCATTAATGATCTCTTCTTCCGTAGGGTGACCTGCGGAAGGATCATTAATGATTGAACGTCTGTCGAGCTTGCTCACAGGCACATCATATCCATAACACCTGTGCACTTGTCGGATGGCTCTAGTGAAGACCGCAAGGTTGGATCTATCCATCTACTTTACATAACAATTCTGTAACAAATGTAGTCTTATTATAACATAATAAAACTTTCAACAACGGATCTCTTGGCTCTCGCATCGATGAAGAACGCAGCGAAATGCGATAAGTAATGTGAATTGCAGAATTCAGTGAATCATCGAATCTTTGAACGCACCTTGCGCTCCTTGGTATTCCGAGGAGCATGCCTGTTTGAGTGTCATGAAAACCCTCAACCCTAGATTGGTTAAAACCTTTCTTTGGTTTGGATTTGGACGTTTGCCGATGATAAGTCGGCTCGTCTTAAAAGTAATAGCTGGATCTGTCTCGCGACATGGTTTGACTTGGCGTAATAAGTATTTCGCTAAGGACATCTTCGGATGGCCGCGTTGCAGGACTAAAGACCGCTTTCTAATCCATTGATCTTACGATTAATATTCTTGACATCTGGCCTCAAATCAGGTAGGACTACCCGCTGAACTTAAGCATATCAATAAGCGGAGGAAAAG
